# Supplementary material for: Are carbon emissions trading and green financial instruments synergistic? -Comprehensive quantitative research based on content analysis
Source: PLoS One. 2024 Mar 7;19(3):e0298601. doi: 10.1371/journal.pone.0298601 (PMC10919601; doi:10.1371/journal.pone.0298601)
Supplement: S1 Data — (ZIP) [file pone.0298601.s001.zip › Data/地级市Z_F工作报告.zip!/║╙─╧/Σ≡║╙╩╨/└┤╫╘╣1⁄2╓┌║┼í╛┬φ┐╦╩2╛▌═°í┐_2009.docx]

各位代表:

现在，我代表市人民政府，向大会作工作报告，请予审议，并请各位政协委员提出意见。

折叠编辑本段工作回顾

2008年，是极不寻常的一年。我们经历了历史罕见、难以预料的重大挑战和考验。在中共漯河市委的坚强领导下，在市人大、市政协的监督支持下，在"新解放、新跨越、新崛起"活动的有力推动下，我们紧紧依靠全市人民，深入贯彻落实科学发展观，积极应对严峻挑战，适时调整工作重点，努力克服各种困难，开拓进取，实干创新，较好地完成了市五届人大三次会议确定的目标任务。

2008年，全市生产总值550.3亿元，增长13.4%;城镇以上固定资产投资213.6亿元，增长33.6%;地方财政一般预算收入19.2亿元，增长15.1%;社会消费品零售总额157亿元，增长22.8%;城镇居民人均可支配收入12364元，增长15.2%;农民人均纯收入5230元，增长14.5%;粮食总产量166万吨，再创历史新高。

(一)工业经济较快增长。全市规模以上工业增加值297亿元，增长19.9%。"重点工业超千亿计划"扎实推进，86家重点企业实现销售收入824亿元、利润93亿元，分别增长17.5%和27.7%。项目建设成果丰硕，市定80个重点项目累计完成投资102亿元，工业"双百工程"年度计划顺利完成，2×30万千瓦热电项目完成投资10.8亿元，金大地100万吨工业盐、双汇5万吨冷库、银鸽15万吨高强瓦楞纸等一批重点项目竣工投产。全市新增规模以上工业企业75家，新增个体私营企业103家;新增中小企业担保机构4家，担保能力达到15亿元。新培育高新技术企业4家，创省名牌产品6个、省优质产品13个，拥有省级以上企业技术中心16个，技术创新和名牌战略取得新成果。

(二)"三农"工作全面加强。认真落实强农惠农政策，发放各项补贴资金2.9亿元。大力发展优质粮食生产，播种面积达到165万亩，国家级农业标准化示范区达到8个。无公害蔬菜基地面积达到56.7万亩，我市成为全省最大的有机蔬菜基地。"双汇产业化工程"深入推进，新培育标准化养殖小区(场)201个，生猪规模饲养比重达到80%，畜牧业增加值占农业增加值的比重突破50%。农业产业化经营水平不断提高，龙头企业发展到382家，农民专业合作社发展到153个，农村土地流转规模25.7万亩。集体林权制度改革走在全省前列，完成造林面积11.6万亩。新农村建设扎实推进，新建改造农村公路283公里，新建户用沼气池4万座，新解决14.8万人饮水安全问题，新建改造农家店376家，新推广各类农机具1237台。新转移农村富余劳动力4.6万人。秸秆禁烧及综合利用工作富有成效，农村环保工作全省领先，"清洁家园"行动初见成效，完成了641个村的村容村貌整治任务，农村生产生活条件明显改善。

(三)改革开放继续深化。企业改革成效明显，一批企业实施了战略重组合作项目。高效行政服务机制建设深入推进，服务型政府建设力度加大。财税、金融改革加快，金融生态环境有所改善。双汇集团与中国银行开展战略合作，获得118亿元授信。漯河商业银行即将挂牌，农信社成功兑付央行票据9.4亿元，银行业不良资产剥离、核销实现突破。对外开放取得新进展。强力实施招商引资"百日会战"，精心组织中国食品名城香港行等大型招商活动，成功举办第六届中国(漯河)食品博览会，签约投资项目67个，总投资190.7亿元，贸易采购合同总成交额165亿元，项目履约率达到80%以上。全年实际利用外资2.4亿美元，实际到位省外资金63亿元，实现进出口总值2亿美元。抢抓国家新增千亿元投资机遇，争取项目资金1.1亿元。

(四)城市建设步伐加快。沙澧河开发建设在全市人民和各个方面的大力支持下，克服了重重困难，取得了重大进展，规划设计高标准完成，安置工作全部结束，"澧河一期"基本竣工，"两桥一闸"进展顺利，"沙河一期"有序展开。城市基础设施日臻完善，人民东路入市口和淞江广场绿化工程、淞江路和海河路升级改造工程全面完成，滦河路、燕山北路建成通车;改造背街小巷21条，新建改造公厕103座，其中二级公厕28座;新铺设供水管网17.3公里、燃气管网63.5公里，新增供水用户9000户、燃气用户1.4万户。京港澳高速公路漯河段改扩建、石武铁路高速客运专线漯河段建设、漯阜铁路电气化改造等工程有序推进。"五城同创"活动扎实开展，城市环境综合整治工作全省领先，顺利通过省级卫生城市复核，国家森林城市建设总体规划通过专家评审，我市荣获中国优秀旅游城市和中国最佳生态旅游城市称号。

(五)社会事业全面发展。以办好省定十大实事和市定十件实事为重点，把保障和改善民生作为工作的出发点和落脚点，投入15.2亿元，着力加强民生工程建设。社会保障体系更加完善，城镇职工养老保险、基本医疗保险覆盖面不断扩大。城镇居民基本医疗保险工作全面推行，企业退休人员养老金标准得到提高。城乡11.5万名特困群众全部纳入低保范围。农村五保户集中供养率达到65%，超过全省平均水平25个百分点。发放个人住房公积金贷款1.8亿元，资金使用率连续五年全省领先。全市新开工建设经济适用房20万平方米，新增廉租户2686户。全民创业活动扎实开展，新增城镇就业5.9万人，超过省定目标2.9万人。下岗失业人员再就业2.5万人，城镇登记失业率低于省控目标2.26个百分点。高等教育得到发展，漯河医专申办本科院校工作启动，漯河职院办学特色明显，漯河食品职业学院申办成功;基础教育得到加强，全部免除了城市义务教育阶段学生学杂费，继续实施"两免一补"政策，大幅度提高农村中小学生人均公用经费标准，校舍建设改造力度加大，学校安全进一步加强。城乡医疗卫生服务体系日趋完善，市传染病医院、市医疗废弃物处置中心和30家社区卫生服务站等项目建成投用，新农合实现了全覆盖。农村信息化建设走在全国前列。沙澧河流域气象中心投入使用。文化、旅游事业繁荣发展，举办了首届市民艺术节，陈星聚纪念馆即将建成，新编豫剧《台北知府》首演成功。全民健身运动深入开展，承办了中美篮球对抗赛等大型体育赛事。计划生育、土地管理和环境保护基本国策得到较好落实。公共机构节能降耗工作走在全省前列。妇女、未成年人、老年人和残疾人的合法权益得到切实保障。统计、广播电视、史志档案、民族宗教、外事侨务、新闻出版等工作都取得新成绩。

(六)社会大局和谐稳定。民主法制和精神文明建设得到加强。633件人大代表议案、建议和政协委员提案全部按时办理完毕。第六届村民委员会换届选举工作顺利完成。法治城市建设成效明显，依法行政水平不断提高。在全省率先开展涉企科室评议活动，审计工作力度加大，纠风治乱成效明显，廉政建设得到加强。社会治安防控体系更加完善，平安建设综合考评全省第一，公共安全感指数全省第二。信访稳定工作取得新成效，我市被评为全省信访工作先进市。应急体系建设进一步加强，安全生产形势持续平稳。食品药品安全监管力度加大、全省领先，有力维护了中国食品名城的声誉。国防教育、双拥共建和人民防空工作继续加强，驻漯解放军、武警部队和民兵预备役人员在抗震救灾和支持地方发展中作出了突出贡献。积极支援四川地震灾区，对口援建工作走在全省前列。

各位代表!在困难形势下能取得这些成绩，是市委正确领导的结果，是人大、政协积极支持的结果，是全市人民共同奋斗的结果，凝聚着各个方面的智慧和力量。在此，我代表市人民政府，向全市广大工人、农民、知识分子、干部和离退休老同志，向驻漯解放军指战员、武警官兵、公安干警以及各界人士致以崇高的敬意!

在肯定成绩的同时，我们也清醒地认识到，伴随宏观环境的重大变化，经济发展中一些深层次的体制性、结构性矛盾进一步凸显，政府工作还存在一些不容忽视的问题。随着国际金融危机逐步加深，市场需求明显不足，部分企业经营困难，经济增速下滑，沙澧河开发建设等重点工程进度受到影响;农民工就业面临困难，农民增收难度加大;第三产业总量较小，水平较低，增速较慢;我市是轻工型产业结构，能耗基数较低，在轻重工业结构调整中，节能减排任务艰巨;一些干部思想观念、工作作风转变不够，少数部门和单位大局意识、发展意识、服务意识不强，发展环境有待进一步优化。对这些问题，我们将以高度的责任感和使命感，采取有力措施，认真加以解决。

折叠编辑本段工作安排

今年，是进入新世纪以来经济发展最为困难的一年，国际金融危机尚未见底，对实体经济的影响进一步加深，困难局面可能进一步加重，在资金供给、财政增收、消费需求、劳动力就业等方面给我们带来了一定压力。对此，我们既要充分认识形势的严峻性，更要充分把握我市应对危机的有利条件和积极因素。一是国家实施的两年"4万亿"投资计划，为我们争取更多的项目和资金，提供了难得机遇。二是我们的主导产业和重点企业运行基本健康，具备一定抵抗风险能力和保持较快发展的基础。我市产业结构以食品、轻工为主，外向度较低，受金融危机的影响相对较轻。三是在金融危机初期，我们快速反应，及时采取应对措施，制定了15条促进经济增长的政策措施，为化解危机争取了主动。四是我市正处于工业化、城市化加速发展阶段，这一阶段的主要特征是二、三产业快速发展，基础设施大量建设，城乡一体化进程不断推进，存在着较大的产业升级空间和巨大的市场需求潜力。五是发达国家和发达地区相关产业将以更快速度向中西部地区梯度转移，我市具备承接转移的区位优势和基础条件，为我们扩大招商引资、承接"南资北移"、进入新行业新领域创造了有利条件。受国际金融危机影响，各种要素价格持续下降，产业、企业整合成本大大降低，关联产业、上下游企业兼并重组步伐进一步加快，我市一些优势企业可以抓住机遇，推动低成本扩张，把比较优势最大限度地转化为竞争优势。六是多年来我们注意牢牢把握投资拉动主战略，大力推动重点项目建设，综合经济实力不断提升，极大地增强了全市人民战胜困难的勇气和力量。特别是在市委的领导下，全市上下思路明晰，认识一致，形成了心齐、气顺、风正、劲足的工作氛围，只要我们坚定信心、咬紧牙关、顶住压力，在困难中大干，在大干中追赶，就一定能够将危机的影响降到最低限度，我们的跨越式发展目标就一定能够实现!

今年经济社会发展的主要目标是:生产总值增长11%，规模以上工业增加值增长15%，地方财政一般预算收入增长9%，全社会固定资产投资增长25%，实际利用外资增长15%，社会消费品零售总额增长14%，城镇居民人均可支配收入增长7%，农民人均纯收入增长7%，单位生产总值能耗、化学需氧量和二氧化硫排放量、人口自然增长率均控制在省定目标以内。

为了实现上述目标，我们必须按照市委五届七次全会要求，深入贯彻落实科学发展观，瞄准"两个率先"目标，着力抓投入、调结构、保增长、惠民生，抢抓发展机遇，创造发展条件，转变发展方式，增强发展动力，确保经济平稳较快发展，确保社会大局和谐稳定。在具体工作中，必须集中力量，突出重点，合力攻坚，务求实现三大突破、三大拉动、三大提升。

(一)强力实施三大突破

实施招商引资大突破。坚持把招商引资作为推动发展的"一号工程"、第一动力，一把手亲自抓;坚持招商与引资并重，既要注重招商，更要注重引资。一要创新方式。建立专业化的招商队伍，实行基地招商、专业招商、以商招商，激活企业招商主体，鼓励返乡农民工创业发展。二要拓宽领域。在突出工业的同时，加大城乡基础设施、农业、商贸、文化、旅游等领域的招商引资力度。三要完善政策。落实好招商引资专项资金和重大项目奖励政策，建立科学的业绩考核体系和经费保障机制，完善正负激励相结合的奖惩办法，对各县区、经济开发区和承担招商引资任务的市直单位，实施严格考核，兑现奖惩，不断调动各级招商引资的积极性。四要突出重点。坚持两腿向外跑项目，依托食品、造纸、盐化工等优势产业，招大、招强、招名牌，瞄准世界500强、国内500强企业，寻求合作发展。坚持两眼向上盯资金，抢抓国家"4万亿"投资机遇，积极争取政策资金。五要注重实效。着力提高招商引资质量和水平，继续强力实施招商引资"百日会战"，举全市之力办好第七届中国(漯河)食品博览会和第四届豫商大会，搭建招商引资平台，力争项目总投资、贸易采购额明显超过往届，签约项目履约率达到80%以上。同时，优化出口商品结构，扩大产品出口规模。

实施产业集聚区发展大突破。紧紧抓住省委、省政府强力推进产业集聚区发展的战略部署，按照整合资源、提升功能、强化特色、增强竞争力的要求，加快集聚区基础设施建设，为工业发展搭建平台。一是科学编制规划。5个省级产业集聚区和2个市级产业集聚区，要按照产城一体、各具特色的原则，抓紧编制规划，明确功能定位，分步推进实施，把产业集聚区建成产业与城区相协调、工业与服务业相协调的示范区。二是加强组织领导。对产业集聚区，实行市级领导干部对口联系工作制度，推行一个产业园区、一套发展规划、一套工作班子、一套政策规定、一套奖惩措施的"五个一"工作法。三是完善政策措施。制定完善园区"飞地经济"政策，引导工业项目向集聚区集中，新增就业人员向园区集中。鼓励产业集聚区采用市场化运作方式，吸引外来资本和民间资金，加快产业集聚区建设，力争早日见到大的成效。四是培育产业特色。依托资源优势和产业优势，突出重点，大力发展食品、煤盐化工、机械、纺织等特色产业，努力使特色产业特色更特、优势更优，形成产业集聚效应。到2012年，全市产业集聚区建成区面积要达到54平方公里，项目总投资850亿元，入驻企业900家，年销售收入超过2400亿元，基本形成规模较大、布局合理、功能完善、特色鲜明的产业集聚区

实施城乡一体化大突破。坚持以城带乡、以工促农，把县城、集聚区、重点镇、中心村作为加快城乡一体化的突破口，推动城乡一体化发展。一是统筹城乡协调发展。市财政安排3000万元支持城乡一体化发展，高标准编制覆盖全市的城乡一体化总体发展规划，特别要制定以市区为核心的100平方公里区域的城乡一体化发展规划，重点抓好"一市两城十镇六线"，努力在部分领域、关键环节实现突破，促进区域协调发展。选定一批试点，制定扶持政策，整合相关资源，从资金、项目等方面重点支持，加快建设一批农村新社区，努力打造出一批亮点，全面推进产业向集聚区集中，人员向城镇、中心村和社区集中，土地向大户集中，养殖业向小区集中，初步形成推进城乡一体化的支撑节点。按照依法、自愿、有偿原则，通过出租、转包、入股等形式，加快农村土地流转，加快发展规模种植和养殖业，为农业规模化、集约化、高效化经营提供广阔空间。切实加强耕地保护，认真开展土地综合整治，统筹安排各类用地，提高土地节约集约利用水平。深化户籍改革，提高城镇综合承载能力，力争城镇化率提高2个百分点以上，让更多的农民过上城镇生活，让更多的农民享受到城乡一体化的发展成果。二是改善农村生产生活条件。建设高标准农田，实行田、水、路、林、电综合治理配套，新打机井4300眼，新增旱涝保收田面积40万亩，改造中低产田3万亩。切实做好秸秆禁烧及综合利用工作。努力提高农业机械化作业水平，积极创建"玉米收获机械化示范市"，力争玉米秸秆还田面积达到75%以上。新修县乡公路200公里、改造农村公路危桥25座以上，新建县级客运站3个。广泛开展农村"清洁家园"行动和生态文明村创建活动，完成400个村的基础设施建设和村容村貌整治工作，实施200个村(镇)绿化工程。特别要抓好107国道、漯舞(阳)、漯北(舞渡)、漯新(店)、漯上(蔡)、漯周(口)等六条公路的沿线环境整治。新解决8万人饮水安全问题，新建规模沼气小区10个、户用沼气池1万座，新建100个连锁超市，新建综合水平较高的示范村35个，新纳入试点村100个以上。切实抓好"村村通宽带工程"，加快自然村通宽带步伐。三是拓宽农民增收渠道。围绕稳粮、增收、强基础、惠民生，全面落实强农惠农政策，大幅度增加农业农村投入，有力促进农业全面发展、农民持续增收。大力发展现代农业，以农业产业化推动农业现代化，整合涉农资金1000万元，加大对龙头企业的扶持力度，新培育市级重点龙头企业20家以上、省级5家以上。全面实施农业标准化生产，抓好粮食核心区建设，打造粮食生产吨粮市。扩大无公害、绿色和有机蔬菜规模，全市蔬菜种植面积达到85万亩。按照"服务城市、致富农村"的思路，发展城郊观光休闲农业。加快生态林业建设，重点沿沙河两岸大力发展花卉、苗木等特色产业，全年完成造林面积11.6万亩，森林覆盖率达到26%。大力发展现代畜牧业，继续实施"双汇产业化工程"，新培育标准化养殖小区20个、规模养殖场100个。四是完善农村服务体系建设。加快建设新型农业科技推广体系，完善农产品质检体系和动物疫病防控体系，不断加快农村信用体系建设，争取创办村镇银行。大力发展农民经纪人队伍，培育发展农民专业合作组织，全市农民专业合作社发展到200家以上。

(二)着力抓好三大拉动

坚持投资拉动。以项目建设为抓手，不断扩大有效投入，强力实施"6113"项目建设计划，突出工业、农林水利、交通能源、城镇基础设施、节能减排和民生改善6个领域，完成重点项目投资100亿元以上、重点工业企业投资100亿元以上，带动全市完成投资300亿元以上。把市定80个重点项目作为投资拉动的重中之重，着力推进2×30万千瓦热电、双汇集团肉鸡产业化、银鸽10万吨文化纸、金大地扩能、永银盐化工、京港澳高速公路漯河段改扩建、石武铁路高速客运专线漯河段建设、漯阜铁路电气化改造等一批重大项目。紧紧抓住国家实施一揽子投资计划的政策机遇，积极谋划一批关联度高、带动能力强的重大项目，争取更多项目纳入国家、省里的投资计划。完善重点项目协调推进机制，坚持市级领导联系分包重点项目和定期观摩制度，全力搞好环境优化和项目服务，加快项目建设。推进金融创新，充分发挥城建投资公司、发展投资公司等五大投融资平台的作用，拓展投融资渠道，通过发行企业债券、扩大信贷投放规模、激活民间资本，着力解决项目建设融资难题。

坚持城市拉动。区域竞争主要是城市的竞争，城市的竞争越来越集中在城市功能、城市形象、城市品牌上，我们要强力实施城市带动战略，搭建平台，提升形象，招引投资，拉动增长。一是突出重点抓特色。集中资源，强力推进沙澧河开发建设，按照"一年一大步、三年大变样"的总体思路，本着"操作要快、效果要好"的原则，坚持决心不减、标准不降，加快建设沙河一期工程，建成太行山路沙河桥、澧河桥和沙河节制闸，规划建设沙澧河交汇处标志性建筑和游园广场，高标准完成生态水域城规划设计，完成下游生态风景林建设，启动沿河重点地域房地产开发，真正把沙澧河开发建设工程打造成精品工程、亮点工程，让沙澧河风景区的美景早日显现，拉动城市品位的提升。二是坚持不懈抓基础。实施嵩山路等市区道路升级改造和整修工程，加大背街小巷、城区公厕改造提升力度。稳步推进城中村改造和旧城改造。加快铁东区域的居住环境改善，规划建设居民休闲场所。开工建设沙北污水处理厂、召陵污水处理厂，完成沙南污水处理厂改造。新建、改造城市供水管网10公里，新铺设燃气中低压管网15公里、供热主支管网35公里，城市供水覆盖率达到90%以上，天然气覆盖率达到70%以上，生活垃圾无害化处理达到100%。强化经营城市理念，推进基础设施投资多元化。三是精益求精抓管理。加大"五城同创"工作力度，一手抓宣传，一手抓治理，不断提高市民的文明意识，开展城市环境综合整治活动，重点解决城乡结合部和铁路沿线的脏乱差问题，加大城区违章建筑治理力度，力争通过国家森林城市验收和省级文明城市评审。完善城市管理办法，提高城市精细化管理水平，加强城市综合执法，提升城市管理的科学化、法制化水平。
